# 

**Published:** 1862-11

**Authors:** 


☞ "HAVING directed the attention to *some* of the defects in the use of language, it becomes proper and necessary, in concluding the subject, to suggest the remedy by which such defects alone can be overcome.

"The first step toward such a desirable consummation *is to cultivate a close and intimate acquaintance with the best writers of every age and every tongue.*"—COSMOS.

Moses and the Prophets! but that's a long STEP—a great deal longer than the two steps the little word "alone," up there, would have to take, to get back to where it belongs in the sentence. W.

### DENTAL REGISTER,

### OF THE WEST.

### Issued Monthly, at $3 a year in advance.

DEVOTED TO THE INTERESTS OF THE DENTAL PROFESSION.

**Edited by J. TAFT and GEO. WATT.**

The Volume commences in January and closes in December.

The Register being issued monthly is always fresh, therefore indispensable to the practitioner who desires to keep posted up with the new inventions and improvements which are daily developed. Neither expense nor effort will be spared to give value and variety to its contents. Articles will be illustrated with well executed engravings, whenever they will add to the interest or assist in explanation.

Its extensive circulation and frequency of issue makes it the BEST DENTAL ADVERTISING MEDIUM in the United States.

☞ CONTRIBUTIONS SOLICITED.

Address Original Communications to **Geo. Watt, Xenia, O.**

Exchanges to **J. Taft, or Dental Register, Cincinnati, Ohio.**

Business Communications to

J. TAFT, Publisher,

56 West Fourth Street, Cincinnati, O.

JNO. T. TOLAND, Proprietor,

38 West Fourth Street, Cincinnati, O.

Communications must reach the Editor as early as the 15th, to insure insertion in the next issue.

